# Induction of Biogenic Magnetization and Redox Control by a Component of the Target of Rapamycin Complex 1 Signaling Pathway

**DOI:** 10.1371/journal.pbio.1001269

**Published:** 2012-02-28

**Authors:** Keiji Nishida, Pamela A. Silver

**Affiliations:** Department of Systems Biology, Harvard Medical School and the Wyss Institute of Biological Inspired Engineering, Harvard University, Boston, Massachusetts, United States of America; University of Glasgow, United Kingdom

## Abstract

Most organisms are simply diamagnetic, while magnetotactic bacteria and migratory animals are among organisms that exploit magnetism. Biogenic magnetization not only is of fundamental interest, but also has industrial potential. However, the key factor(s) that enable biogenic magnetization in coordination with other cellular functions and metabolism remain unknown. To address the requirements for induction and the application of synthetic bio-magnetism, we explored the creation of magnetism in a simple model organism. Cell magnetization was first observed by attraction towards a magnet when normally diamagnetic yeast *Saccharomyces cerevisiae* were grown with ferric citrate. The magnetization was further enhanced by genetic modification of iron homeostasis and introduction of ferritin. The acquired magnetizable properties enabled the cells to be attracted to a magnet, and be trapped by a magnetic column. Superconducting quantum interference device (SQUID) magnetometry confirmed and quantitatively characterized the acquired paramagnetism. Electron microscopy and energy-dispersive X-ray spectroscopy showed electron-dense iron-containing aggregates within the magnetized cells. Magnetization-based screening of gene knockouts identified Tco89p, a component of TORC1 (Target of rapamycin complex 1), as important for magnetization; loss of *TCO89* and treatment with rapamycin reduced magnetization in a *TCO89*-dependent manner. The *TCO89* expression level positively correlated with magnetization, enabling inducible magnetization. Several carbon metabolism genes were also shown to affect magnetization. Redox mediators indicated that *TCO89* alters the intracellular redox to an oxidized state in a dose-dependent manner. Taken together, we demonstrated that synthetic induction of magnetization is possible and that the key factors are local redox control through carbon metabolism and iron supply.

## Author Summary

Most organisms do not respond to magnetic fields. However, “magnetotactic” bacteria and migratory animals can sense geomagnetic fields and alter their behavior accordingly. These organisms often contain small magnetic particles that may be responsible for sensing magnetic fields. In magnetotactic bacteria, specific genes are crucial for the formation of these magnetic particles, but no such genes have yet been characterized in migratory animals. In humans, formation of magnetic particles can be observed in the neuronal tissue in neurodegenerative diseases. One explanation for the appearance of these magnetic particles is that they are the result of alterations in metabolism, which occur in neurodegenerative diseases. Here, we explore this hypothesis by inducing magnetism in yeast cells, which are not naturally magnetic and examine how changes in metabolism contribute to particle formation and magnetism. We find that yeast cells expressing a set of human proteins that sequester iron contain iron particles and become attracted by a magnet when grown with ferric citrate. Through physiological and genetic studies we show that target of rapamycin complex 1 (TORC1) signaling, which responds to nutritional signals, is important for the magnetization of these cells by altering the intracellular oxidation (or redox) state. We also show that genes involved in carbon metabolism affect magnetization. We propose that local redox control mediated by carbon metabolism and iron homeostasis, processes that exist in normal unmagnetized cells, are key for iron particle formation and magnetization. We conclude that magnetization of normal cells will be possible with these existing gene sets.

## Introduction

In biology, magnetism is a unique and virtually orthogonal physical property. As magnetic interactions can be contactless, remote, and permeable, integration of magnetic properties into biological systems provides another dimension for bioengineering and therapy. Magnetic functions may provide a unique interface between cells; for example, magnetic sensing as an input and induced magnetization as an output would allow not only magnetic manipulation but also magnetometric readout such as magnetic resonance imaging (MRI). Only a few natural systems are known to exploit magnetic function. Magnetotactic bacteria produce a chain of organelles called magnetosomes [1], in which ferromagnetic magnetite (Fe3O4) or greigite (Fe3S4) particles are formed (reviewed in [2]). The cells orient and swim along geomagnetic field lines locating better growth conditions more efficiently than random swimming (reviewed in [3]). In the genomes of these bacterial species, specific clusters of genes called magnetosome gene islands are conserved [2]. The recent comprehensive study has revealed several specific genes participating in various steps of formation of the magnetosome [4], showing the complexity of biogenesis of the organelle. A putative iron transporter gene MagA from magnetotactic bacteria [5] has been shown to be sufficient for producing MRI-detectable iron-containing particles in mammalian cells [6],[7]. However, MagA gene does not belong to the magnetosome gene island and the MagA protein localizes to the plasma membrane in *Magnetospirillum magneticum* strain AMB-1 [8],[9]. To date, no other successful transgenic study for magnetosomal function has been reported. Members of magnetotactic bacteria identified so far belong to α-proteobacteria, δ-proteobacteria, *Nitrospira* (reviewed in [2]), and γ-proteobacteria [10], while intracellular magnetic inclusions were also found in *Shewanella putrefaciens* [11] and a photosynthetic purple bacteria [12] and a geo-biological study has presented magneto-fossils that are too large for bacteria—a possible remnant of eukaryotic biogenic magnetic particles [13].

Migratory animals sense geomagnetic fields, an ability called magneto-reception (reviewed in [14]). The radical pair and the magnetite hypothesis are the two proposed modes for the mechanism of magneto-reception. The former may exploit a photochemical reaction affected by the magnetic field, and the latter involves small magnetic particles in the nervous system. To date, neither physiological nor molecular mechanisms for the formation of such magnetic particles in nerve tissue are understood.

However, anomalous deposition of iron is found to be associated with many neurodegenerative disorders in humans, such as Alzheimer's, Parkinson's, and Huntington's disease (reviewed in [15],[16]). Being an essential element for life, iron is reactive and prone to precipitate under physiological conditions. Some modification of iron homeostasis is assumed to induce iron mineralization in nerve tissue. Recently, an iron-export ferroxidase activity of β-amyloid precursor protein [17] was identified, indicating the importance of iron homeostasis in nerve tissue integrity. Owing to its ability to catalyze the formation of reactive oxygen radical species, high concentration of free iron ions could thus be toxic. Cells may deal with this by producing ferritin, a ubiquitous iron sequestrating protein present from bacteria to human [18]. Ferritin oligomerizes to form a shell of 12–15 nm in diameter in which iron is sequestered and mineralized. Iron mishandling by ferritin causes neurodegeneration with iron deposition called neuroferritinopathy [19]. Naturally isolated ferritin from horse spleen contains paramagnetic ferrihydrite (5Fe2O3•9H2O), and every single core (8–10 nm at most) is in principle too small to be manipulated magnetically. However, the composition may vary depending on the chemical environment as magnetite-formation of ferritin was demonstrated in vitro [20] exhibiting superparamagnetic behavior at room temperature.

Here, we address these issues by exploring the synthetic induction of bio-magnetization using a model organism budding yeast *Saccharomyces cerevisiae*.

## Results

### Ferritin Complements Iron Tolerance of Yeast Deficient in Vacuolar Iron Sequestration

**Figure 1:**
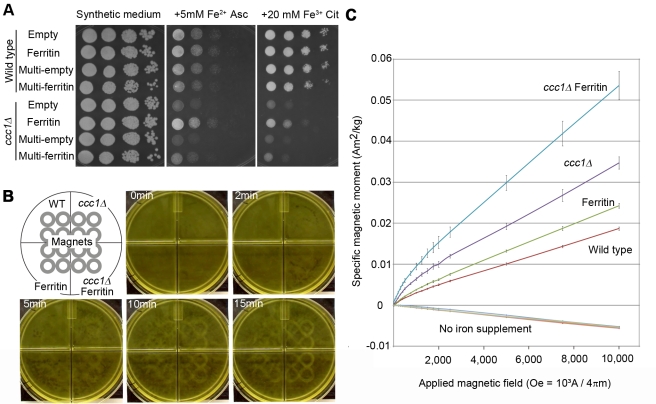
Magnetization of yeast. (A) Iron tolerance affected by *ccc1Δ*, ferritin, and Fe^2+^/Fe^3+^. Culture of 2.0 OD^600^ was diluted 10^2^, 10^3^, 10^4^, and 10^5^-fold in water, spotted by 10 ul from left to right for each strain, and grown on indicated plates. Empty, single-copy empty plasmid-harboring strain; Ferritin, single-copy ferritin gene set plasmid-harboring strain; Multi-empty, multi-copy empty plasmid-harboring strain; Multi-ferritin, multi-copy ferritin gene set plasmid-harboring strain. (B) Cell attraction towards magnet. Cell suspensions were placed over magnets (4×4 gray circles) and a picture taken at indicated times. Corresponding video is Video S1. (C) SQUID magnetization curve. Induced specific magnetic moment was measured as a function of applied magnetic field. Lines for the four strains without ferric citrate supplementation (No iron supplement) are almost identical and overlapped; mean ± s.d. (*n* = 3).

For biogenic magnetization, significant amounts of magnetic compounds need to be formed inside the organism. This may be achieved by altering iron homeostasis either physiologically or genetically. Since ferrous iron is prone to oxidation to insoluble ferric iron, citric acid, a chelator of the ferric ion, can be included to prevent precipitation with no impact on biological availability. Wild-type yeast cells can grow at as high as 5 mM ferrous (Fe^2+^) ascorbate or 20 mM ferric (Fe^3+^) citrate (Figure 1A, wild type). Ferric citrate was less toxic and can thus be used to deliver iron to yeast without damaging the cells or forming precipitates in the media.

Yeast cells lack ferritin and sequester iron in their vacuoles. The vacuolar iron transporter Ccc1p plays a major role in iron sequestration, loss of which abolishes iron tolerance [21]. Human ferritin genes consist of ferritin heavy chain FTH, ferritin light chain FTL, and the iron chaperone PCBP1 [22]. As described previously, the ccc1 knockout strain (*ccc1Δ*) showed intolerance at 5 mM ferrous ion while as high as 20 mM ferric citrate is required to see intolerance of *ccc1Δ* (Figure 1A), suggesting mitigated iron toxicity of ferric citrate. We found that single copy expression in yeast of the human ferritin gene set conferred iron tolerance to *ccc1Δ* both in ferrous and ferric supplements (Figure 1A), indicating that ferritin efficiently sequesters iron in these conditions.

### Altered Iron Homeostasis and Ferritin Confer Magnetism to the Cell

The four strains (wild type containing empty plasmid, ferritin-expressor, *ccc1Δ*, and *ccc1Δ* ferritin-expressor) were cultured in 20 mM ferric citrate liquid medium and tested for magnetization. The cell cultures were exposed to magnets and attraction was observed. Attraction of *ccc1Δ* ferritin-expressor was detectable as early as 2 min after exposure. After 10 min, attraction of all strains became observable (Figure 1B and Video S1).

For quantitative characterization of the magnetic properties of the yeast cells, a superconducting quantum interference device (SQUID) was used. The cells were subjected to a measurement of their magnetic moment at 300 K at various magnetic fields to analyze field-dependent magnetization. Without ferric citrate supplementation all the four strains similarly exhibited negative values proportional to the applied field (Figure 1C, no iron supplemented), indicating that they are diamagnetic. As is the case for most biological materials, their mass magnetic susceptibility (m^3^•kg^−1^) was comparable to that of water (−9.051×10−9) (Table 1). When supplemented with ferric citrate, all the strains exhibited positive values. At high fields (2,500 to 10,000 Oe), magnetization is proportional to field and not saturating, indicating a dominant contribution of paramagnetism. At low fields (0 to 2,000 Oe), an upward concave curve of magnetization was observed, indicating additional ferro/ferri-magnetic contribution, which typically saturates within this region. This suggests that the cells contain mostly paramagnetic (or superparamagnetic) material with a slight amount of ferro/ferri-magnetic material. Mass magnetic susceptibility of the paramagnetic constituent was given based on values at high fields (Table 1). Those of ferritin-expressor, *ccc1Δ*, and *ccc1Δ* ferritin-expressor were approximately 1.3, 1.8, and 2.8 times larger than that of wild type, respectively. Previous studies on magnetic susceptibility of isolated ferritin ranged from 3.7×10^−8^ to 9.4×10^−8^ m3/kg (originally 2.95×10^−6^ to 7.5×10^−6^ em in cgs unit) at room temperature, depending on the sample and measuring method [23] (reviewed in [24]). Thus, we observed a gain of magnetic susceptibility due to ferritin expression, while a non-ferritin contribution was also present, indicating that ferric citrate supplementation induces basal magnetization in yeast. *ccc1Δ* showed increased magnetization compared to wild type, suggesting that non-vacuolar iron may have more magnetic contribution than previously thought. The synergistic effect of ferritin and *ccc1Δ* can be explained by higher availability of iron to ferritin in the cytosol.

**Table 1:** Mass magnetic susceptibility of ferric citrate–supplemented cells.

χ_mass_ (10^−8^)	WT	Ferritin	*ccc1Δ*	*ccc1Δ*+Ferritin
Normal	−0.694	−0.700	−0.702	−0.673
+ iron	2.14	2.79	3.80	5.98

Mass magnetic susceptibility (χ_mass_ m^3^/kg) was given by an equation χ_mass_ = 4π10^−3^s/*H*. s is the specific magnetic moment (emu/g or Am^2^/kg) and H is magnetic field (Oe or 10^3^A/4πm). Values of 2,500 and 10,000 Oe were applied.

### Magnetized Cells Contain Electron-Dense Deposition within Membranous Structures

**Figure 2:**
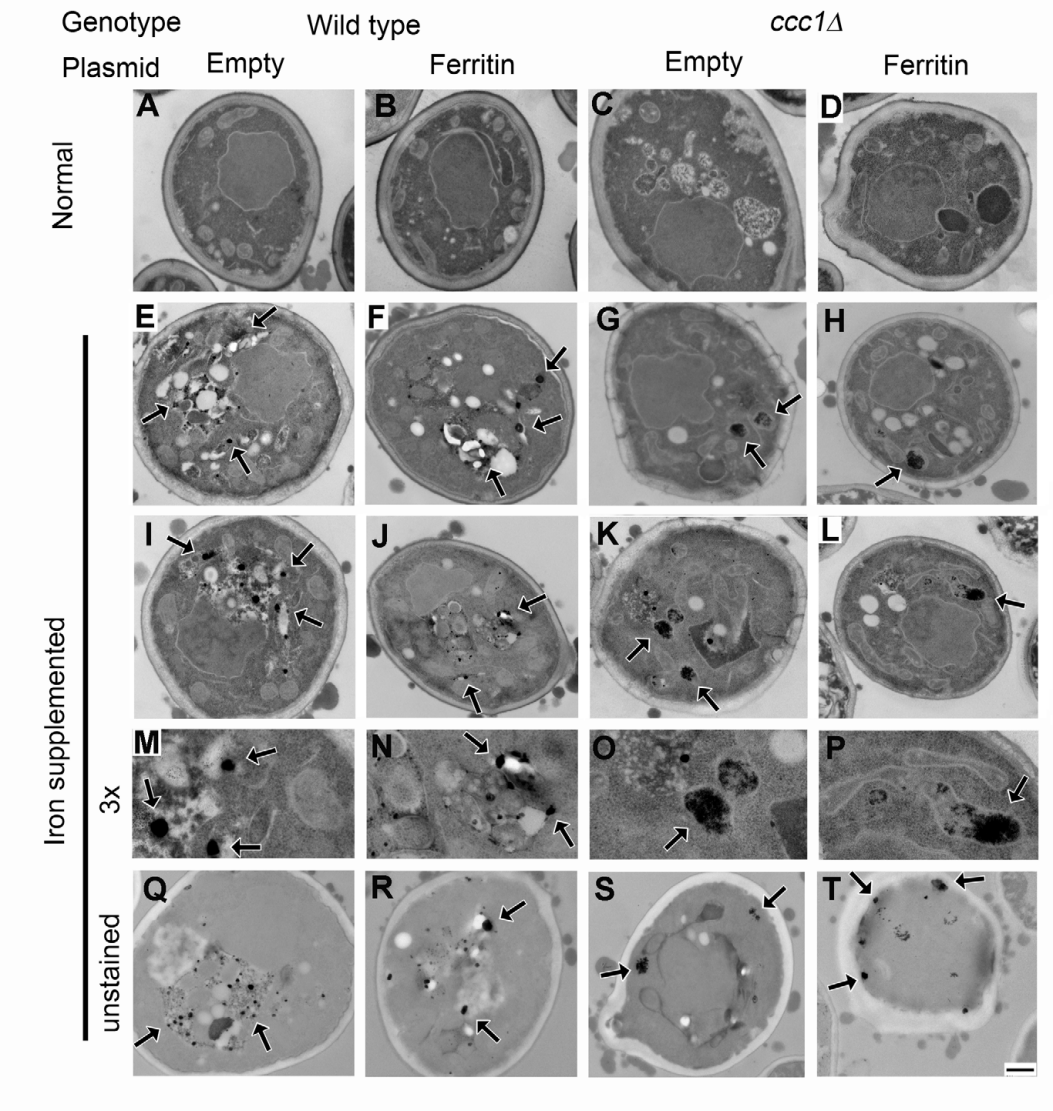
Electron micrographs of magnetized cell. Normally grown cells (A–D) and iron-supplemented cells (E–T) of wild-type (A, E, I, M, and Q), ferritin-expressor (B, F, J, N, and R), *ccc1Δ* (C, G, K, O, and S), and *ccc1Δ* ferritin-expressor (D, H, L, P, and T) are shown. (M–P) 3× magnification of (I–L), respectively. Unstained sections are shown in (Q–T). Arrows indicate electron-dense particles. Scale bar, 500 nm.

Ultrathin section transmission electron microscopy showed accumulation of electron-dense deposits (Figure 2). Although these varied in shape, size, and amount among cells, wild type cells typically contained round particles associated with membranous structures that are most likely the vacuoles (Figure 2 wild-type), while *ccc1Δ* cells tended to contain aggregates within mitochondria (Figure 2 *ccc1Δ*). As the mitochondria are where cells convert inorganic iron into heme and iron-sulfur clusters, the observed deposits could be caused by iron overload due to the defect in vacuolar iron sequestration, and may contribute to the higher magnetic susceptibility in *ccc1Δ*. Ferritin expression had little observable effect on the electron micrographs (Figure 2 ferritin) perhaps due to the small size of the iron binding center.

### Electron-Dense Deposits Contain Iron and Phosphorous

**Figure 3:**
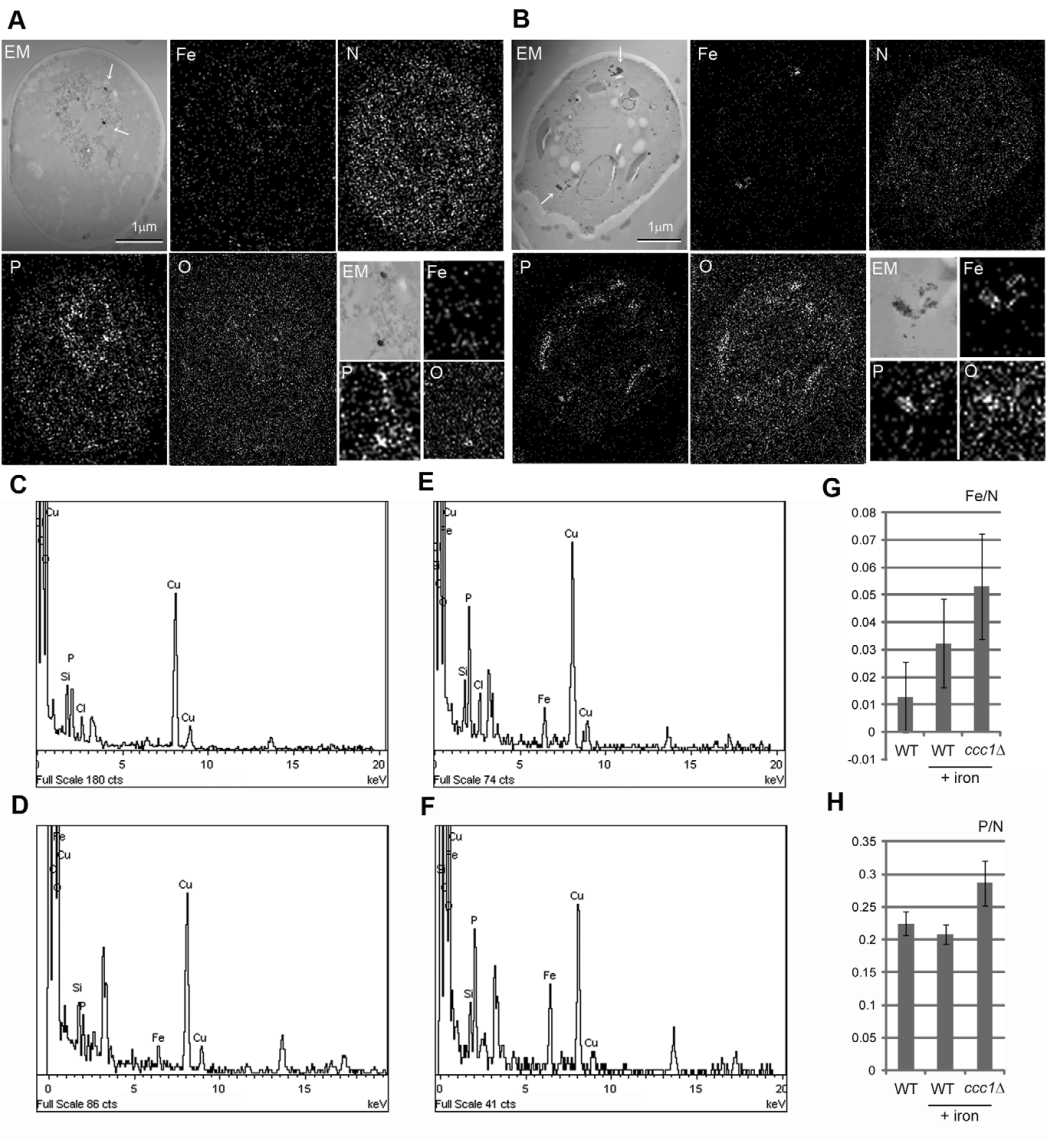
EDS elemental analysis for magnetized cell. Wild type (A) and *ccc1Δ* (B) are analyzed for counter image of dark-field electron micrograph (EM), iron (Fe), nitrogen (N), phosphorous (P), and oxygen (O). Arrows indicate electron-dense deposits. Two-fold magnifications are shown on the bottom-right. EDS spectra are shown for cytoplasmic region of wild type (C) and *ccc1Δ* (D) and electro-dense particles of wild type (E) and *ccc1Δ* (F), respectively. Vertical axis is for counts and horizontal axis is for energy (keV). Note copper peaks are from EM grid. Unassigned peaks are presumably from uranium used for staining. Relative element counts of iron (G) and phosphorous (H) versus nitrogen are calculated for sectioned whole cell region of normally grown wild type, iron-supplemented (+iron) wild type, and *ccc1Δ*.

To reveal the elemental composition of the electron-dense deposits, magnetized cells were analyzed by energy-dispersive X-ray spectroscopy (EDS). Elemental maps were obtained for detectable elements, and iron, phosphorous, oxygen, and nitrogen showed characteristic distributions associated with cellular structures (Figure 3A and 3B). Nitrogen was distributed throughout the cell consistent with association with biogenic molecules such as proteins. In wild type cells grown in ferric citrate, phosphorous, iron, and oxygen were slightly concentrated within membranous structures presumably vacuoles and in electron-dense round particles (Figure 3A, magnified images). In ccc1Δ cell, iron showed increased localization to the clusters of electron-dense crystals (Figure 3B). Phosphorous also accumulated in the clusters. Oxygen showed a similar pattern to phosphorous with less contrast. These two types of electron-dense deposits (small round particles in wild type and clustered crystals in *ccc1Δ*) thus contained iron, oxygen, and phosphorous with different composition stoichiometries (Figure 3E and 3F). The elemental maps were further analyzed to estimate relative amounts of iron and phosphorous (Figure 3G and 3H); iron was higher in *ccc1Δ* than in wild type cells, whereas phosphrous showed only a small increase in *ccc1Δ*.

### Cells Can Be Trapped by a Magnetic Column

**Figure 4:**
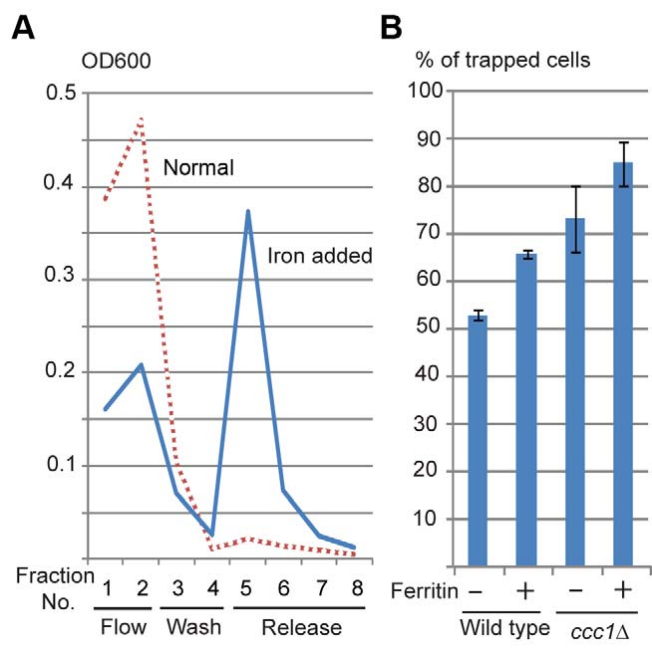
Magnetizable column entrapment. (A) Wild type cells grown in normal medium (Normal) and in 20 mM ferric citrate (iron supplemented) were applied to magnetizable column, and fractions of flow-through (Flow), wash-out (Wash), and trapped cells released after magnet detachment (Released) were collected and monitored by OD^600^. (B) Magnetic entrapment efficiency of the four strains. Percentages of trapped cells were calculated; mean ± s.d. (*n* = 3).

Magnetic columns have been used for separation of biomaterials labeled with magnetic particles. To test if our yeast cells behave similarly, the cells were applied to a magnetic column. Normally grown yeast cells were not retained on the column under the conditions tested (Figure 4A, normal). The cells supplemented with ferric citrate were retained by the magnetized column (Figure 4A and 4B). Among the four strains, the order of rate of trapped cells is in agreement with their magnetic susceptibility measured by SQUID, indicating that this system can be used for comparison of cell magnetization, as well as to separate magnetic cells.

### A Component of TORC1 Is Important for the Magnetization

**Figure 5:**
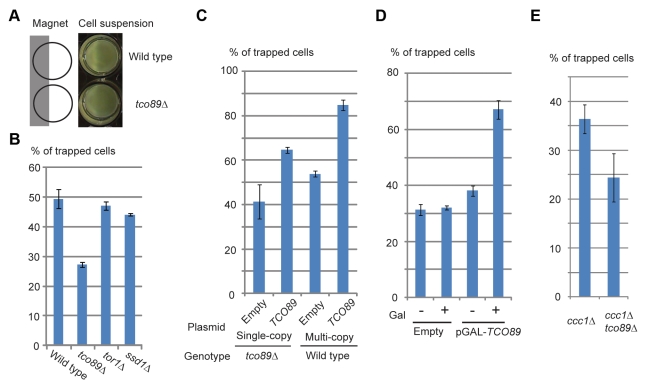
Effect of *TCO89* on magnetization. (A) Reduced magnetic attraction of *tco89Δ*. Cell suspensions were adjusted to 0.5 OD^600^, attached to magnet at half of the bottom, and let stand for 30 min. (B–E) Magnetizable column entrapments were performed as in Figure 3B. (B) TOR pathway gene knockouts. (C) Effect of *TCO89* copy number. Strains harboring indicated plasmids were grown in selection medium to keep plasmid. (D) Induction of *TCO89* expression under galactose inducible *GAL1* promoter. *tco89Δ* cells harboring indicated plasmid were pre-cultured in raffinose medium containing 10 mM ferric citrate and transferred to raffinose (–) or galactose (+) medium containing 10 mM ferric citrate. (E) Genetic interaction between *CCC1* and *TCO89*. *ccc1Δ* and *ccc1Δtco89Δ* double knockouts were grown in synthetic complete medium containing 10 mM ferric citrate.

Genetic control of magnetization would greatly expand the engineering potential of magnetic cells. To explore this possibility as well as to gain further insight into the nature of the biogenic magnetization, we sought yeast gene knockout strains that show altered magnetization. Candidate genes to be tested were selected based on their functional or phenotypic description associated with iron homeostasis or oxidative stress from *Saccharomyces* Genome Database (http://www.yeastgenome.org). Mutant strains were grown in 20 mM ferric citrate medium and their attraction towards a magnet was observed (Figure 5A). Strains showing reproducible altered attraction were selected and subjected to magnetic column separation to confirm and quantify their magnetization. From the initial screen of 60 strains (Table S1), *tco89Δ* was found to show consistent reduction of magnetization compared to wild type (Figure 5A and 5B). Tco89p is known to be a nonessential component of TORC1 [25]. TORC1 globally regulates cell growth in response to nutrient, stress, and redox states (reviewed in [26], [27]). To ask if and how TORC1 is involved in the magnetization, Tor1p, the other nonessential component of TORC1 and Ssd1p, which coordinates with TORC1 to maintain cell integrity [25], was tested. Both *tor1Δ* and *ssd1Δ* showed little change in the magnetization (Figure 5B), indicating that challenged cell integrity is not associated with the magnetization. Magnetization of the cells positively correlated with copy number of *TCO89* (Figure 5C), showing a dose-dependent effect of *TCO89* on the magnetization. Expressing *TCO89* under a galactose inducible promoter pGal1 showed induction of magnetization (Figure 5D).

### Magnetization by TCO89 Is Independent from CCC1 But Dependent on TORC1 Activity

**Figure 6:**
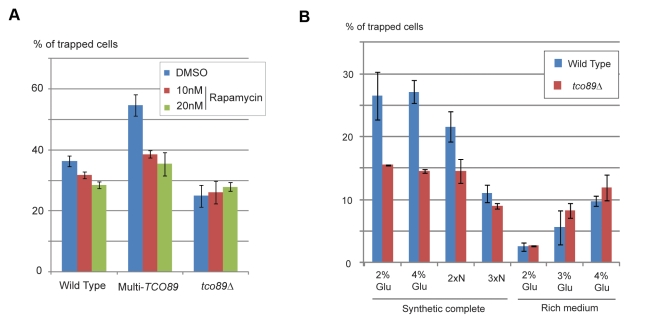
Effects of TORC1 activity on magnetization. (A)Wild type harboring empty plasmid (Wild type), harboring multi-copy TCO89 plasmid (Multi-*TCO89*), and *tco89Δ* harboring empty plasmid (*tco89Δ*) were grown in selection medium containing 10 mM ferric citrate and 10 nM (red column), 20 nM (green column) rapamycin, or 0.2% dimethyl sulfoxide (DMSO) (blue column) as control. (B) Wild type (blue column) and *tco89Δ* (red column) are pre-cultured in synthetic complete medium and grown in indicated media containing 10 mM ferric citrate. Indications of media are synthetic complete medium containing 2% or 3% glucose (2% Glu, 3%Glu), 2- or 3-fold concentration of amino acid supplements (2×N, 3×N), or rich medium containing 2%, 3%, or 4% glucose (2% Glu, 3%Glu, and 4%Glu), respectively. Magnetization was measured as in Figure 3B.

Loss of *TCO89* in *ccc1Δ* decreased magnetization (Figure 5E), suggesting that iron sequestration into the vacuole by *CCC1* does not have a predominant effect on induction of magnetization by *TCO89*. In contrast, *TCO89* affects magnetism through TORC1 activity. We used rapamycin, an inhibitor for TORC1 at sub-lethal doses. Rapamycin treatment reduced magnetism in wild type and more prominently in multi-copy *TCO89*, while no reduction is observed in *tco89Δ* (Figure 6A), indicating that induction of magnetism by *TCO89* is through TORC1 activity.

### Carbon/Nitrogen Balance Affects Magnetism

As TORC1 processes nutritional signals, we tested if the nutritional environment affects magnetism. Compared to synthetic-defined medium, we observed a reduction of magnetism when cells were grown in rich medium with the same amount of iron (Figure 6B). The magnetism then increased as extra glucose was added to rich medium. In contrast, addition of extra nitrogen (i.e., amino acids and nucleotides) to synthetic defined medium decreased magnetism, suggesting that the relative availability of carbon and nitrogen has impact on the formation of magnetism. Effect of *TCO89* became less prominent in rich medium or when extra nitrogen was added. These results indicate that higher carbon availability has a positive effect on magnetization, which is enhanced by *TOC89*.

### *TCO89* Controls Redox State

**Figure 7:**
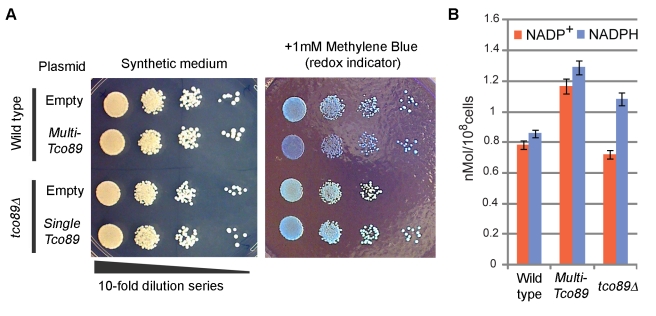
Redox control by *TCO89*. (A) Dose-dependent effect of *TCO89* on cell redox state. Culture of 2.0 OD^600^ was diluted 10^2^, 10^3^, 10^4^, and 10^5^-fold in water, spotted by 10 ul from left to right for each strain, and grown on indicated plates for 2 d, and colony staining by methylene blue was observed. Bluer color indicates more oxidized. (B) Cellular NADP measurement. NADP^+^ (red column) and NADPH (blue column) were extracted from cells grown in liquid medium, measured, normalized by cell number, and plotted; mean ± s.d. (*n* = 3).

As iron homeostasis has a close relationship with redox state, we asked if *TCO89* has any function associated with redox control. Cellular redox activity can be monitored by a biocompatible redox indicator methylene blue, which loses its color when reduced. Equal numbers of cells were spotted and grown on plates containing methylene blue to observe colony staining. Compared to wild-type or plasmid-complemented cells, *tco89Δ* exhibited little color while multi-copy *TCO89* cells were blue (Figure 7A), indicating that *TCO89* leads cellular redox to an oxidized state in a dose-dependent manner. *tco89Δ* also showed compromised cell growth in the presence of methylene blue presumably because a higher rate of methylene blue reduction interferes with cellular metabolism.

Nicotinamide adenine dinucleotide phosphate (NADP) is a coenzyme that serves as a redox mediator in protection against oxidative stress. Cells harboring multi-copy *TCO89* showed higher levels of both NADP^+^ (oxidized) and NADPH (reduced), while *tco89Δ* had slightly lower NADP^+^ and higher NADPH (Figure 7B).

### Genes for Carbon Metabolism and Mitochondrial Redox Affect Magnetism

We expanded genetic screening for candidates related to carbon metabolism and redox using the magnetic column entrapment procedure (Figure S1). Gene knockouts affecting oxidative damage, such as GRX2, GRX3, and SOD2, did not show significant changes, while POS5, a gene for mitochondrial NADH kinase, showed reduction in magnetism. In contrast, UTR1 did not affect magnetism, which is a cytoplasmic ATP-NADH kinase. Gain of magnetism was seen with loss of YFH1, which has been reported to accumulate iron in mitochondria [28],[29], and whose human homolog FXN is responsible for the neurodegenerative disease Friedreich's ataxia [30]. Regarding carbon metabolism, gene knockouts for SNF1 and ZWF1 showed reduction in magnetism. SNF1 is required for processing carbon stress signals (reviewed in [31]) and ZWF1 codes for an enzyme at the branch point of the pentose phosphate pathway [32].

## Discussion

We demonstrated that generation of bio-magnetization in yeast is possible by three ways: modulating iron homeostasis, introducing iron crystallizing proteins, and controlling redox state. Intracellular redox state is normally sufficiently reductive to allow iron to exist as soluble Fe^2+^. Oxidation of Fe^2+^ to Fe^3+^ facilitated by *TCO89* led to an oxidative state that induces iron precipitation and yeast magnetization.

The importance of redox state in magnetization offers insight into magnetotactic bacteria. Most of these bacteria thrive exclusively in microaerobic environments, showing their strong preference to certain redox conditions. Evolutionally, formation of bio-magnetic particles may have originated as a consequence of redox mediation and/or iron sequestration, although today's magnetosomes seem so specified to magnetic function that once formed they cannot be utilized as resources by the cell [3],[33]. In the gradient of oxygen/iron distribution in deep aqueous environments, there may exist cells adapted to certain redox and chemical conditions that were optimal for the formation of magnetite or greigite [34]. In such cells magnetization and redox metabolism could have been linked. Once magneto-aerotaxis was established, the role of iron as a redox mediator became less important as seen in today's magnetotactic bacteria.

Although TORC1 activity has been suggested to be redox-sensitive [35],[36], its redox control has not been demonstrated. Considering TORC1 function in the regulation of carbon metabolism and energy production, which are major sources of redox flux, *TCO89* may control redox state through these functions. Indeed, carbon availability showed a significant impact on magnetism. In glucose-rich conditions, glycolysis and fermentation is preferred over mitochondrial TCA cycle and oxidative phosphorylation. As the TCA cycle generates reducing equivalents that then reduce oxygen, down-regulation of mitochondrial TCA cycle may result in a shift to a more oxidized state inside mitochondria. In agreement, recent studies have detected ferric phosphate nano-particles in mitochondria from fermenting yeast [37],[38]. Combined with the results that iron deposits were found in mitochondria of *ccc1Δ*, oxidizing conditions in mitochondria could facilitate iron deposition that has greater magnetism. Iron deposition in neurodegeneration may also be attributed to a failure in mitochondrial redox control in concert with energy metabolism because of the brain's high demand for energy and oxygen.

Redox control may also aid in applications that involve biogenic metal precipitation such as bioremediation and nano-particle production. Because our choice of organism was not based on any potential of magnetization of yeast, these ideas could be easily applied to a number of other organisms to confer them with similar or possibly greater properties, or to find key components for redox control.

## Materials and Methods

### Strains and Culture Conditions

Constructs were made via a BioBrick assembly method [39],[40]. Ferritin genes, which consist of FTL, FTH1, and Pcbp1, were obtained from ATCC mammalian gene collection. The open reading frames of the genes were PCR-amplified and assembled under control of the CCC1 promoter from yeast. The genes were then cloned into yeast single-copy plasmid pRS316 or multi-copy plasmid pRS416 [41]. Plasmids were transformed into BY4741 (*MATa his3Δ1 leu2Δ0 lys2Δ0 ura3Δ0*) or *ccc1Δ* (*his3Δ1 leu2Δ0 lys2Δ0 ura3Δ0 ccc1::kanMX4*). All knockout strains were obtained from Saccharomyces Genome Deletion Project [42] that have BY4741 background with gene deletion by kanMX4. *TCO89* gene including promoter and terminator region was PCR amplified and cloned into pRS316 or pRS426. For galactose induction, *TCO89* without promoter region was cloned under pGAL1 in pRS316. Cells were grown in synthetic medium (0.67% yeast nitrogen base) or synthetic dropout medium (0.67% yeast nitrogen base without amino acid, 0.2% dropout supplements) with appropriate carbon source (2% glucose, 2% raffinose, or 2% galactose) at 30°C. Ferric citrate and ferrous ascorbate were freshly added from 1 M stock solutions. Methylene blue was added from 10 mM stock solution.

### Attraction Test

Each strain was pre-cultured in synthetic medium and then diluted into the medium supplemented with 20 mM iron citrate and grown overnight. The cells were collected by centrifugation and re-suspended to give 0.5 OD^600^. Five ml of the suspensions were layered onto 1 ml Optiprep density gradient medium (Axis-Shield PoC AS, Norway) in four-compartmented Petri dishes. Each dish was placed over a black paper sheet and axial pole magnets R848 (K&J Magnetics, Inc. PA) aligned 4×4 reciprocally. For magnetic screening, 0.9 ml cell suspension was layered on 0.1 ml Optiprep density gradient medium in 24-well flat bottom plate. Block magnet BZ084 was attached from the bottom of the plate.

### Transmission Electron Microscopy

Cells were chemically fixed and embedded as described previously [43],[44]. Ultrathin sections (60–80 nm) were cut on a Reichert Ultracut-S microtome, placed onto copper grids, and stained with 0.2% lead citrate. Non-stained sections were also prepared to avoid staining artifacts. Specimens were examined on a JEOL 1200EX-80 kV transmission electron microscope and images were acquired with DITABIS digital imaging plates.

### EDS Analysis

Non-stained sections same as for transmission electron microscopy were analyzed on JEOL JEM 2010F at 200 kV with JEOL Dark field STEM detector (probe size 1.0 nm, camera length 15 cm). EDS analysis was performed by INCA system (Oxford Instruments, UK).

### SQUID Magnetometry

Cells were collected by centrifugation, washed twice with 0.6 M sorbitol, dehydrated in −20°C acetone, and freeze-dried. Dried samples were encapsulated and weighted. Direct-current field-dependent SQUID magnetometry was performed at 300 K from 0 to 10,000 Oe (10^3^ A/4 πm) using Quantum Design AC and DC Magnetic Property Measurement System. Specific magnetic moment was given by Am^2^/kg.

### Magnetic Column Entrapment

Cells were collected by centrifugation, suspended in ST (0.6 M sorbitol containing 0.01% Triton-X 100), incubated for 30 min at room temperature, and adjusted to 0.5 OD^600^. MACS cell separation MS columns (Miltenyi Biotec Inc., CA) were placed in a ring magnet R848, equilibrated with ST, loaded with 1 ml cell suspension, washed by 1 m ST, then displaced from the magnet, and bound cells were released by 1 ml ST. The unbound fraction (flow-through and wash-out) and trapped fraction were measured by OD^600^ and the percentage of trapped cells was calculated.

### NADP Measurement

Cells were grown in synthetic medium and harvested at OD^600^ 0.6–0.7. Each culture containing about 10^8^ cells was sampled, left to stand for 10 min, spun down at 2,400 rpm for 5 min, cooled on ice, washed with ice-cold PBS plus 0.01% Triton-X100, and subjected to extraction and detection using Fluoro NADP/NADPH (Cell Technology, CA) following the manufacturer's instruction. Fluorescence was measured at 540 nm excitation and 590 nm emission in Wallac 1420 Multilabel counter (PerkinElmer, Finland).

## Supporting Information

Figure S1 Magnetic column screening for redox and carbon metabolism genes. Knockout strains were grown in synthetic complete medium containing 5 mM ferric citrate and measured for magnetization as in Figure 3B.
(TIF)

Table S1 **Candidate knockout strains subjected to magnetic screening.**
(DOC)

Video S1 **Cell attraction towards magnet. 16× fast-forwarded video for pictures of** **Figure 1B** **.**
(WMV)

## Abbreviations

EDS: energy-dispersive X-ray spectroscopy
NADP: nicotinamide adenine dinucleotide phosphate
SQUID: superconducting quantum interference device
TORC1: target of apamycin complex 1
