# Supplementary figures and images for: Induction of Biogenic Magnetization and Redox Control by a Component of the Target of Rapamycin Complex 1 Signaling Pathway
Source: PLoS Biol. 2012 Feb 28;10(2):e1001269. doi: 10.1371/journal.pbio.1001269 (PMC3289596; doi:10.1371/journal.pbio.1001269)

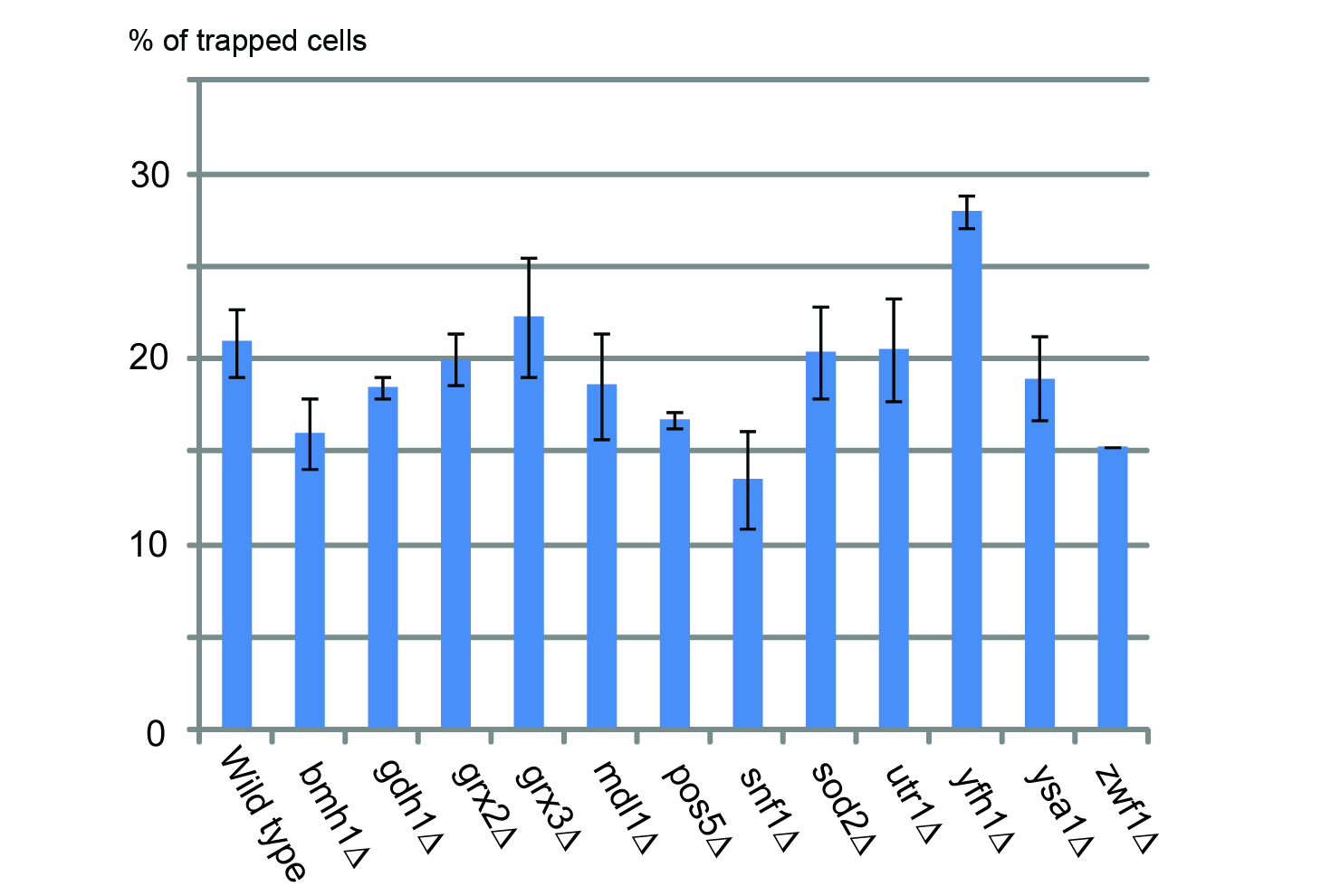

Supplement: Figure S1 — Magnetic column screening for redox and carbon metabolism genes. Knockout strains were grown in synthetic complete medium containing 5 mM ferric citrate and measured for magnetization as in Figure 3B. (TIF) [file pbio.1001269.s001.tif]
