# Supplementary material for: Induction of Biogenic Magnetization and Redox Control by a Component of the Target of Rapamycin Complex 1 Signaling Pathway
Source: PLoS Biol. 2012 Feb 28;10(2):e1001269. doi: 10.1371/journal.pbio.1001269 (PMC3289596; doi:10.1371/journal.pbio.1001269)
Supplement: Table S1 — Candidate knockout strains subjected to magnetic screening. (DOC) [file pbio.1001269.s002.doc]

**Supplementary Table**. Candidate knockout strains subjected to magnetic screening

| *gtt2* | *grx3* | *tsa1* | *cta1* | *grx1* | *ccs1* | *alo1* | *yig1* | *tma19* | *ybl055c* |
| --- | --- | --- | --- | --- | --- | --- | --- | --- | --- |
| *trx1* | *ycf1* | *mdm30* | *msn5* | *trx3* | *cyc2* | *ybp1* | *tim18* | *mdl1* | *ysa1* |
| *yap1* | *gto1* | *prx1* | *ccp1* | *gsh1* | *trr2* | *alf1* | *tco89* | *fcp1* | *bmh1* |
| *fre3* | *trx2* | *ctt1* | *gtt1* | *grx4* | *trx3* | *sod2* | *gln3* | *uth1* | *hyr1* |
| *gsh2* | *skn7* | *glo4* | *sod1* | *ftr1* | *grx2* | *aft1* | *ecl1* | *frm2* | *tor1* |
| *fth1* | *fre1* | *glr1* | *pbi2* | *fet3* | *ybp2* | *lot6* | *mrpl25* | *mep2* | *fet4* |

All the strains derived from *BY4741* and corresponding genes are replaced by *kanMX* marker.
